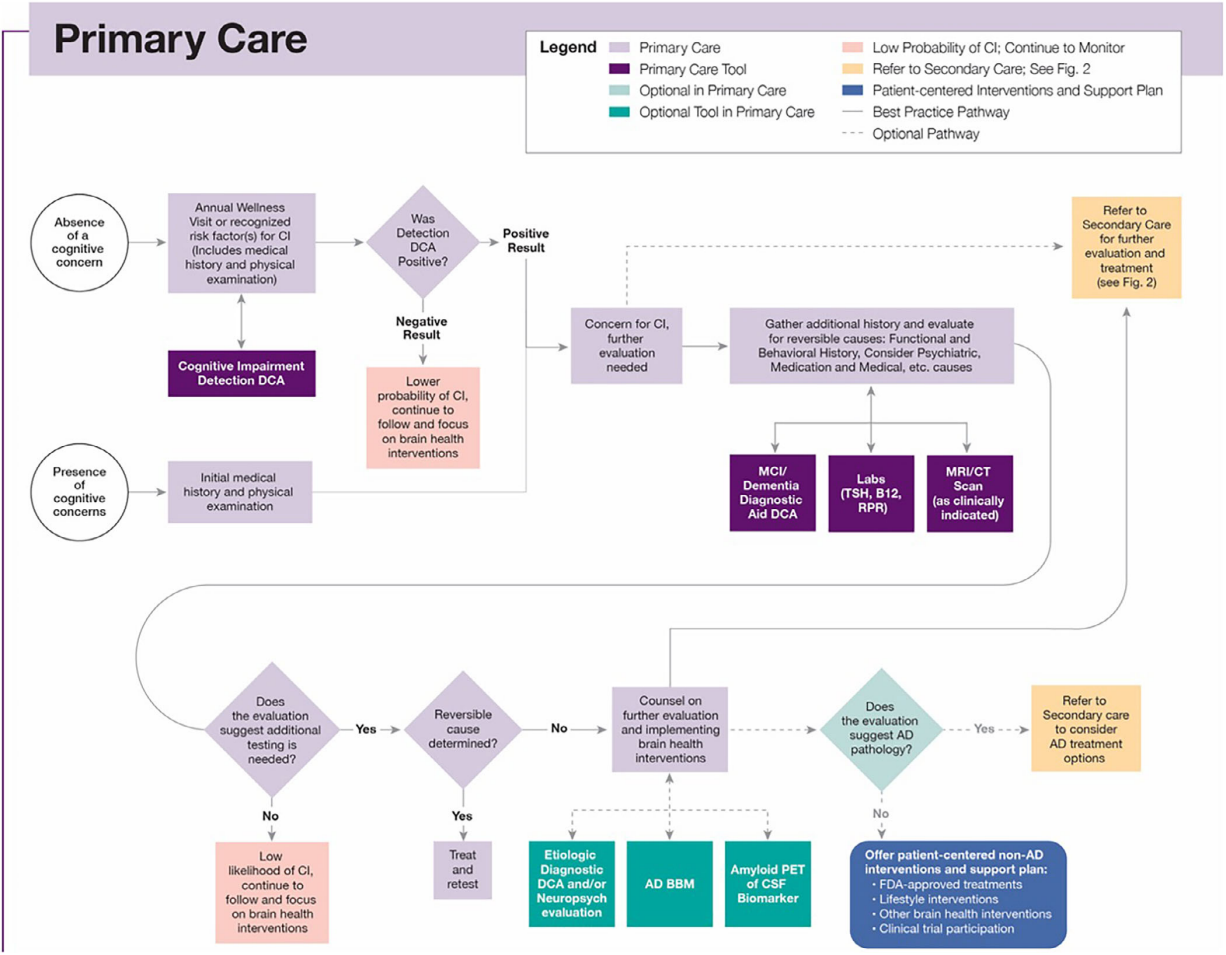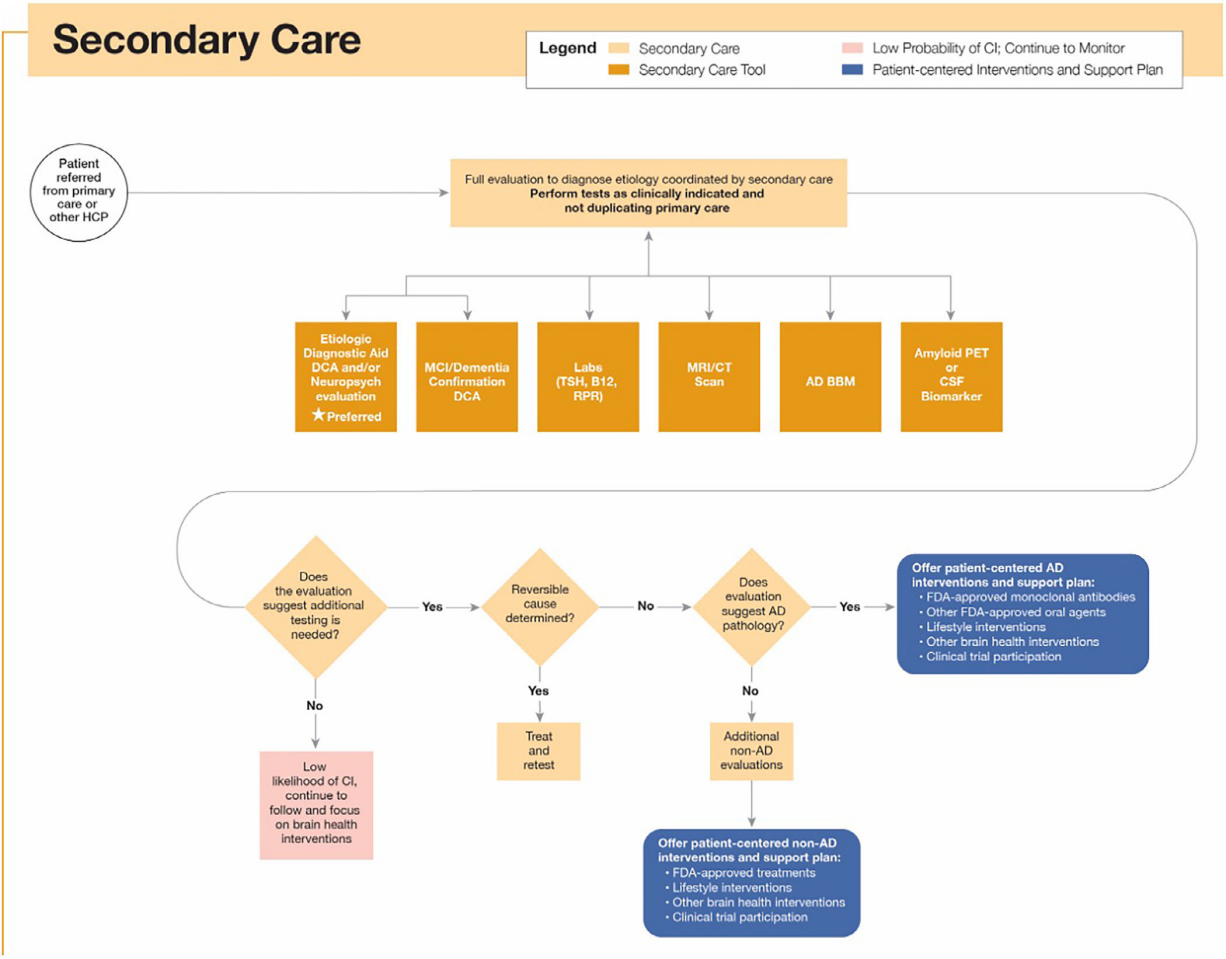# Advancing Clinical Adoption of Digital Cognitive Assessments: Implementation Pathways to Improve Early Detection and Diagnosis of Cognitive Impairment

**DOI:** 10.1002/alz70857_103023

**Published:** 2025-12-25

**Authors:** Pierre N. Tariot, Melissa Petersen, Darren R. Gitelman, Ishtar Govia

**Affiliations:** ^1^ Banner Alzheimer's Institute and University of Arizona College of Medicine, Phoenix, AZ, USA; ^2^ Institute for Translational Research, University of North Texas Health Science Center, Fort Worth, TX, USA; ^3^ Advocate Health, Downers Grove, IL, USA; ^4^ Amagi Health, London, United Kingdom

## Abstract

**Background:**

Early detection of cognitive impairment (CI) is critical for identifying individuals with or at risk for Alzheimer's disease (AD), enabling timely interventions that can improve care, enhance quality of life, and potentially slow disease progression. However, traditional paper‐based cognitive assessments face limitations, e.g., lengthy administration times, weak psychometric properties, subjective administration and scoring, and limited accessibility. These barriers can delay diagnosis and intervention, underscoring the need for scalable, cost‐effective, psychometrically sound, and time‐efficient tools. Digital cognitive assessments (DCAs) promise improved accuracy, reduced testing times, and lower cost. To realize these benefits and support clinical adoption, a standardized framework for DCA implementation is essential.

**Method:**

The Global CEO Initiative on Alzheimer's Disease (CEOi) convened a multidisciplinary workgroup consisting of experts in research, clinical practice, industry, and patient advocacy. The goal is to advance the adoption of DCAs in clinical care, enabling a faster and more accurate diagnosis. The primary objective is to develop implementation pathways including understanding the key facilitators and barriers to integrating DCAs into healthcare workflows.

**Result:**

Clinical implementation pathways were developed for three contexts of use: detection of possible CI absent a recognized concern; confirmatory aid to diagnosing mild cognitive impairment or dementia when a cognitive concern is raised by a patient, caregiver, or healthcare provider; and supporting the evaluation of a neurodegenerative etiology in cases of confirmed CI, including assessment for AD. The recommended pathways integrate DCAs into primary and secondary care settings, offering flexible workflows to accommodate varying capabilities of health systems and practices. This framework is designed to streamline adoption, enhance diagnostic accuracy, and ensure DCAs are adaptable to clinical contexts. These pathways emphasize a holistic approach, where decisions are informed by cumulative data and guided by clinician judgment.

**Conclusion:**

DCAs have the potential to transform detection and diagnosis by overcoming the limitations of traditional tools. The Workgroup's defined use cases and implementation pathways establish a strong foundation for clinical adoption, advancing global efforts in brain health. Continued multistakeholder collaboration and expert‐driven consensus can lead to full realization of the impact of DCAs in improving detection and diagnosis and the pragmatic considerations for their implementation.